# The Theory of Counter Irritation

**Published:** 1873-12

**Authors:** James Ross

**Affiliations:** Manchester.


### ARTICLE XII.

### *The Theory of Counter-Irritation.*

BY JAMES ROSS, M. D, MANCHESTER.

Counter-irritation was defined as the application of an irritant to one part of the body in order to influence morbid action in its vicinity. The theory advanced was that (1) the influence of the counter-irritant is conveyed by continuous and contiguous tissue, and not through the bloodvessels and the nerves; and (2) the influence conveyed is always of a stimulant character. An endeavor was made to deduce the first position from the general theory of inflammation; and the author stated that the second assumption would account for all the effects which counter-irritants are known to produce in the treatment of various diseases. A stimulant action might aggravate the disease in the first stage of inflammation, and counter-irritants were known to produce this effect occasionally. At other times a stimulant action might in this stage assist the disease through its natural progress, by developing the second stage of inflammation. An instance of this effect occurred when the pain of pleurisy was relieved by a blister. In such a case the disease was not checked, but the effusion separated the pleuræ, and the pain was relieved. In the second stage of inflammation, and especially in chronic cases, a stimulant action was most likely to promote health, and it was in such cases that counter-irritants were most safely employed. A similar remark might be made with regard to cases of local debility, in the treatment of which counter-irritants were found useful. Quantitative differences were found to exist in the effects of counter-irritants according, first, to the proximity of the irritant to the seat of the primary disease, and, secondly, to the degree of the artificial irritation produced, and these differences were easily explicable on the supposition that the influence exerted by the counter-irritant upon the disease was of a stimulant nature.—*Medical and Surgical Reporter.*

